# Supplementary figures and images for: The level of putative carotenoid-binding proteins determines the body color in two species of endemic Lake Baikal amphipods
Source: PeerJ. 2020 Jun 19;8:e9387. doi: 10.7717/peerj.9387 (PMC7307558; doi:10.7717/peerj.9387)

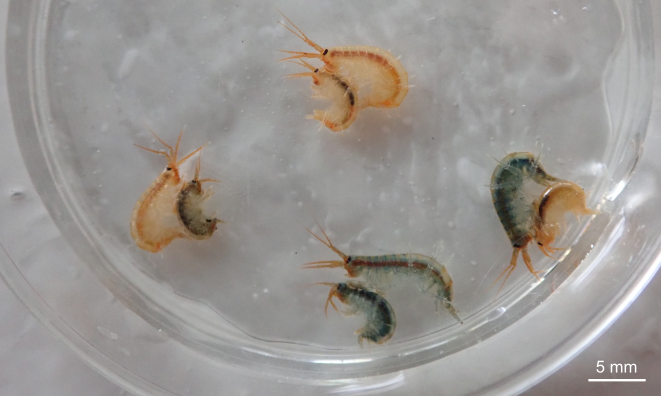

Supplement: Supplemental Information 1 — All animals originate from the same catch. [file peerj-08-9387-s001.png]

**A**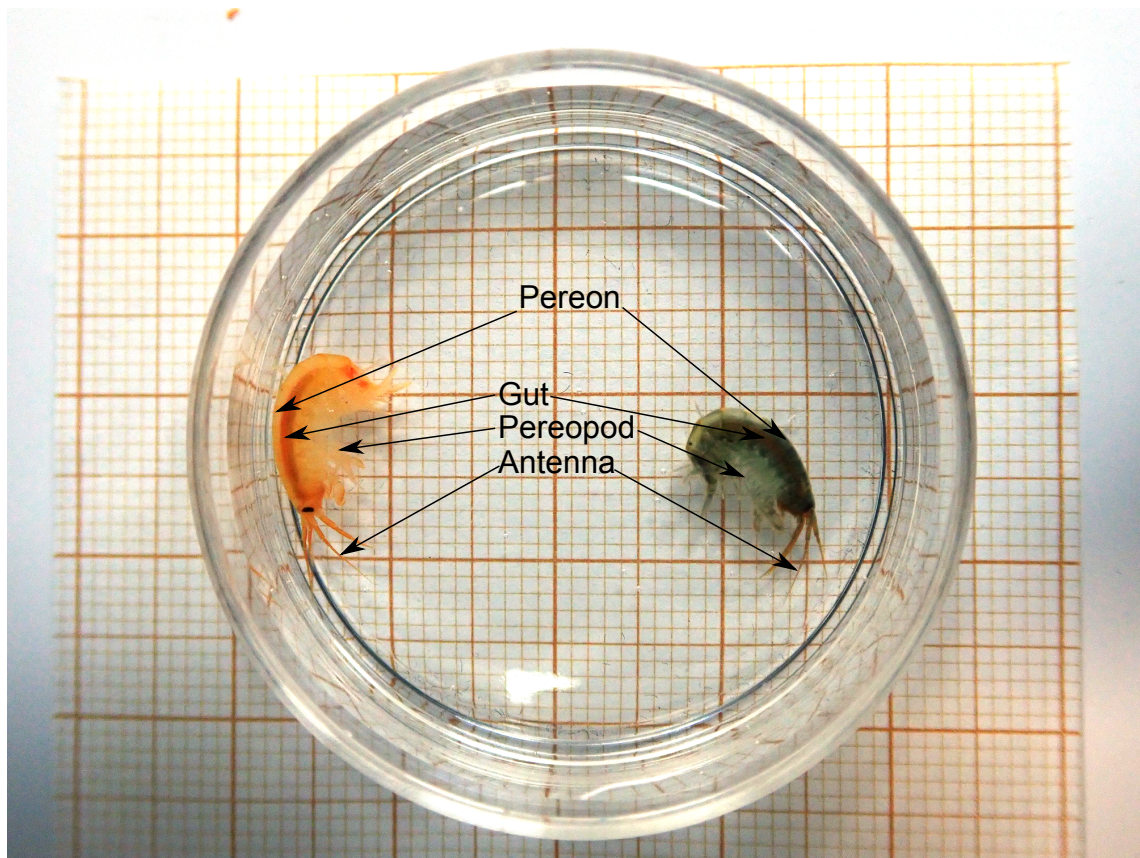**B**

Gut R/B color index

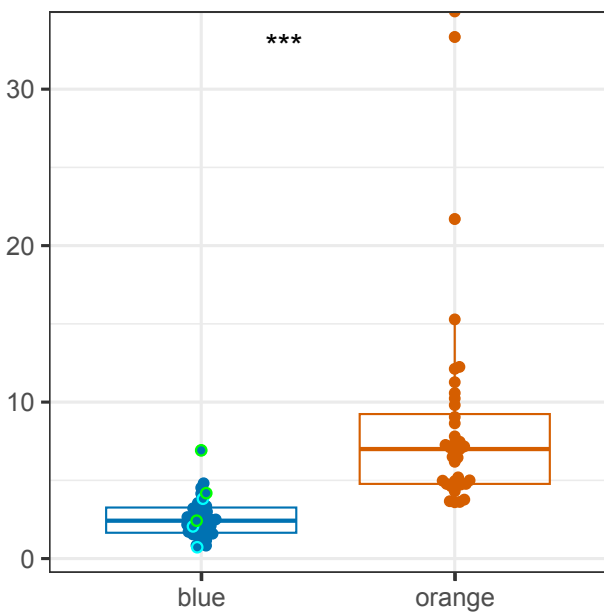**C**

Pereopod R/B color index

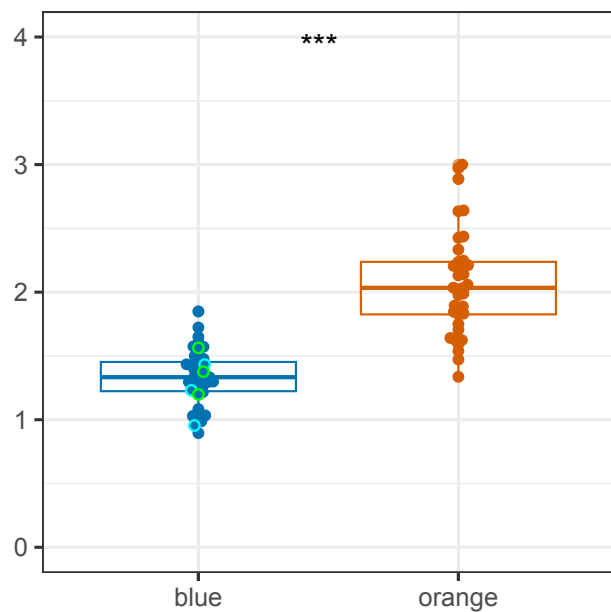

Supplement: Supplemental Information 2 — (A) Example of a photograph used for color quantification. (B) Gut color index. (C) Pereopod color index. See Fig. 2 for color codes. [file peerj-08-9387-s002.pdf]

*E. cyaneus*

2DE\_1 (orange)

2DE\_2 (blue)

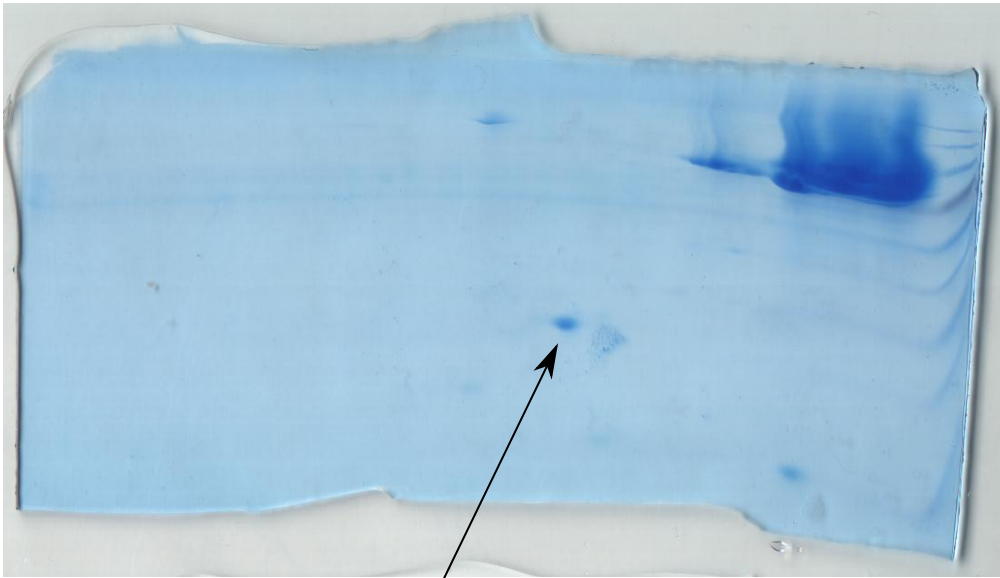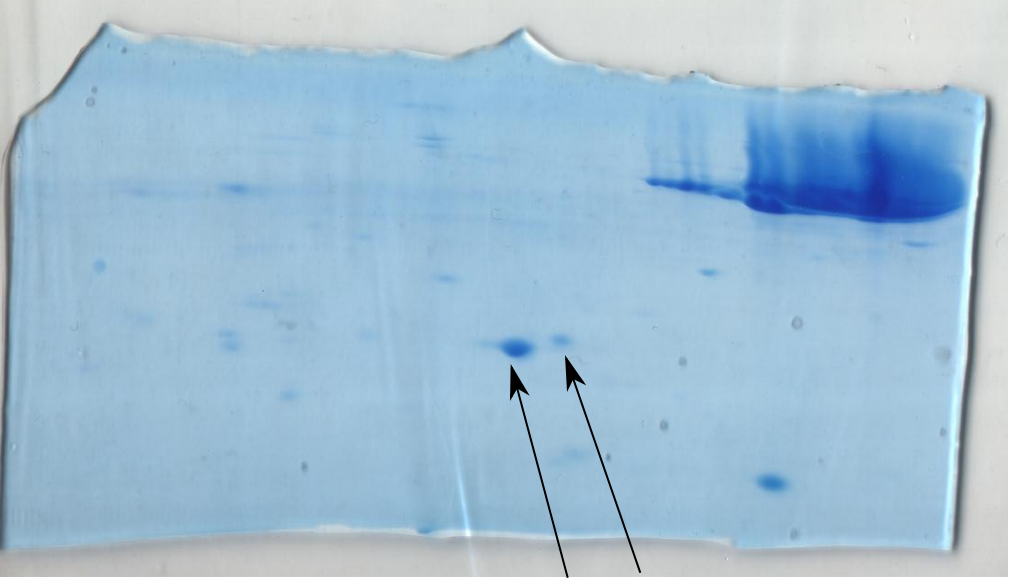

pI 3

2DE\_1.27kD\_a

pI 3

2DE\_1.27kD\_a / b

126 129 134 133 136 137 138 139 140

115 116 117 118 99 96 121 122 125

2DE\_1 2DE\_2  
o b

Ld#5 127 168 172 189 Ld#2  
1ul 6ul

70  
55  
40  
35  
25  
15

*E. vittatus*

Teal

Yellow

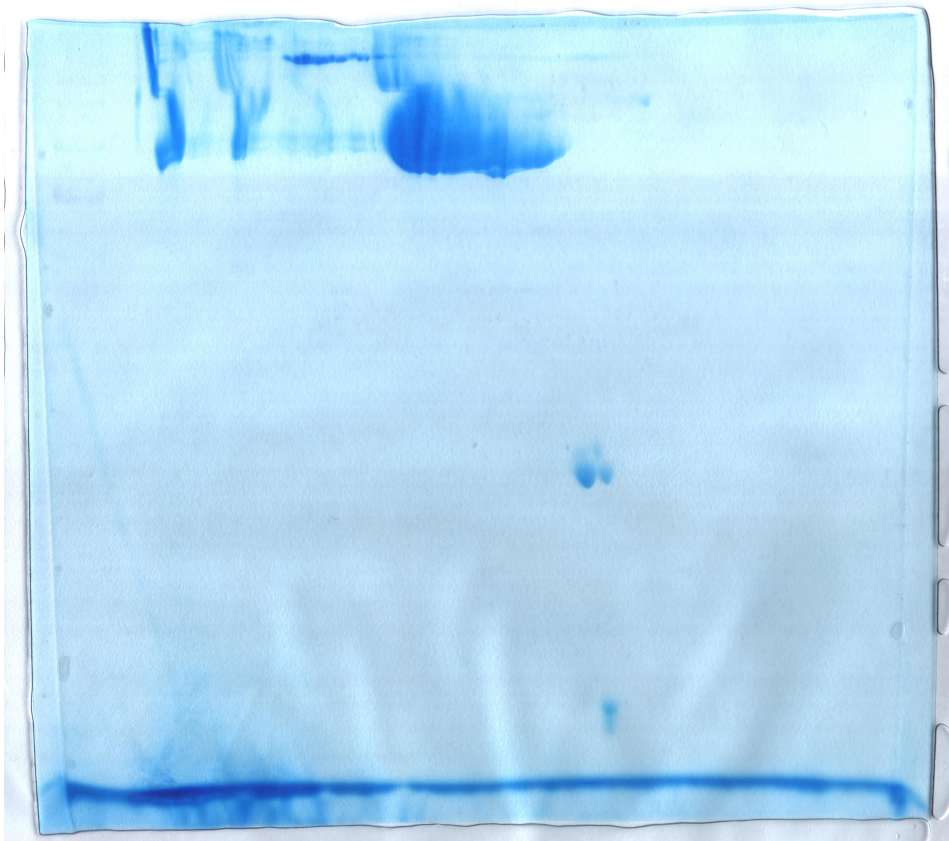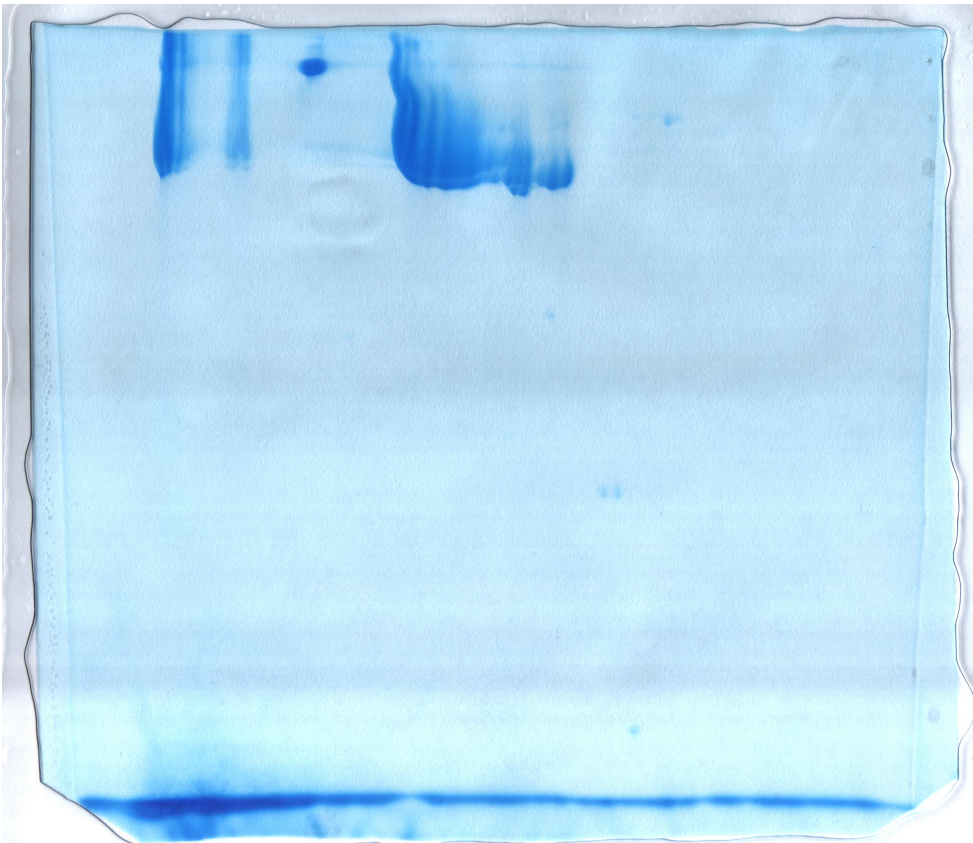

Supplement: Supplemental Information 3 [file peerj-08-9387-s003.pdf]

**A**

Correlation coefficient = 0.96

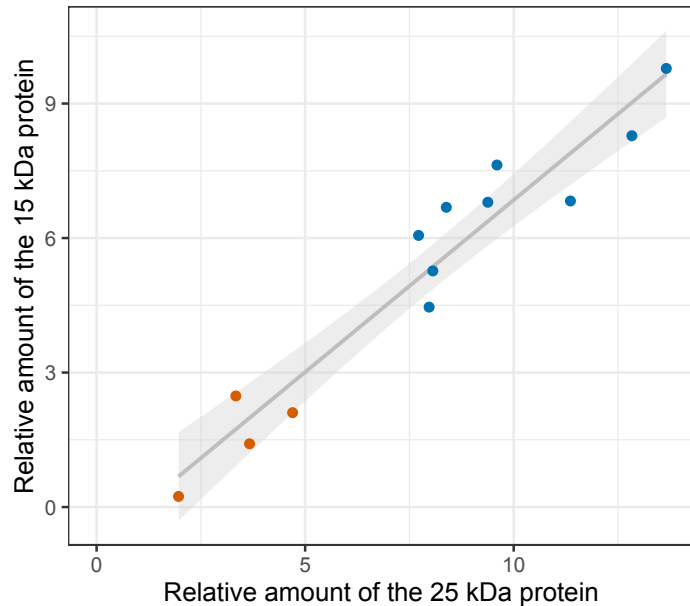**B**

Correlation coefficient = 0.97

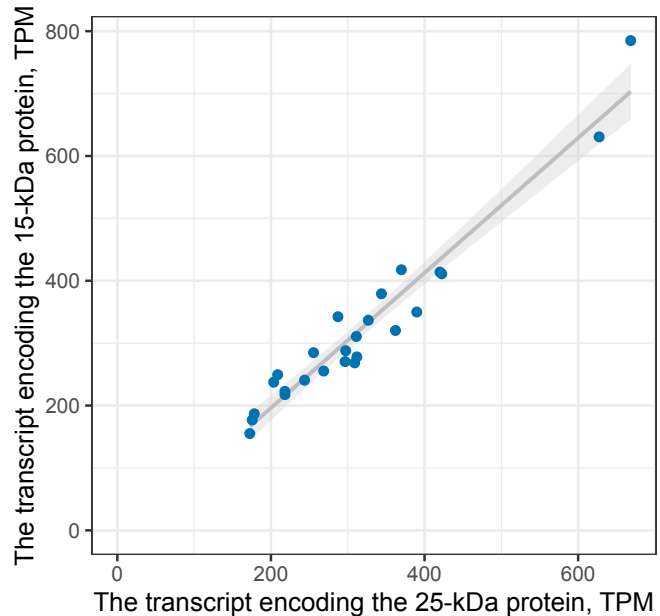

Supplement: Supplemental Information 4 [file peerj-08-9387-s004.pdf]

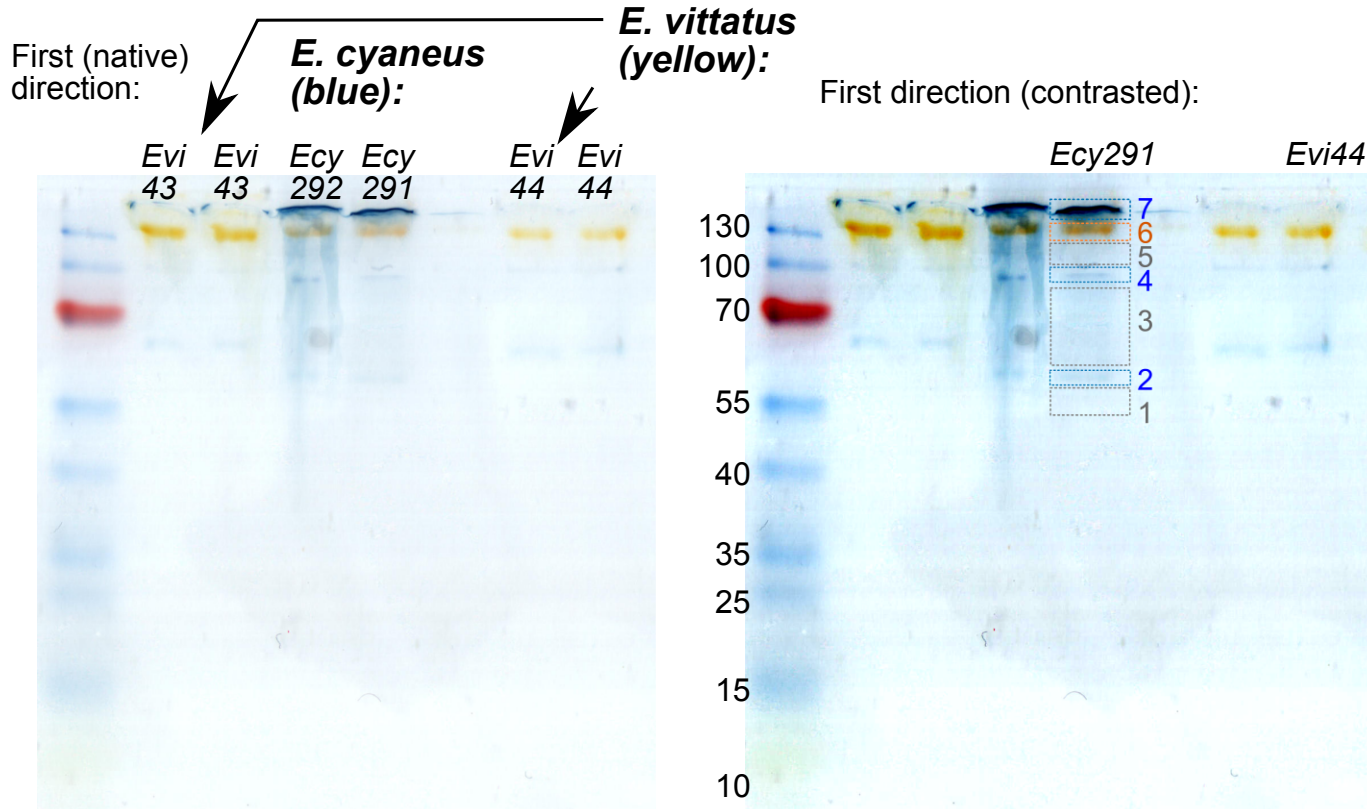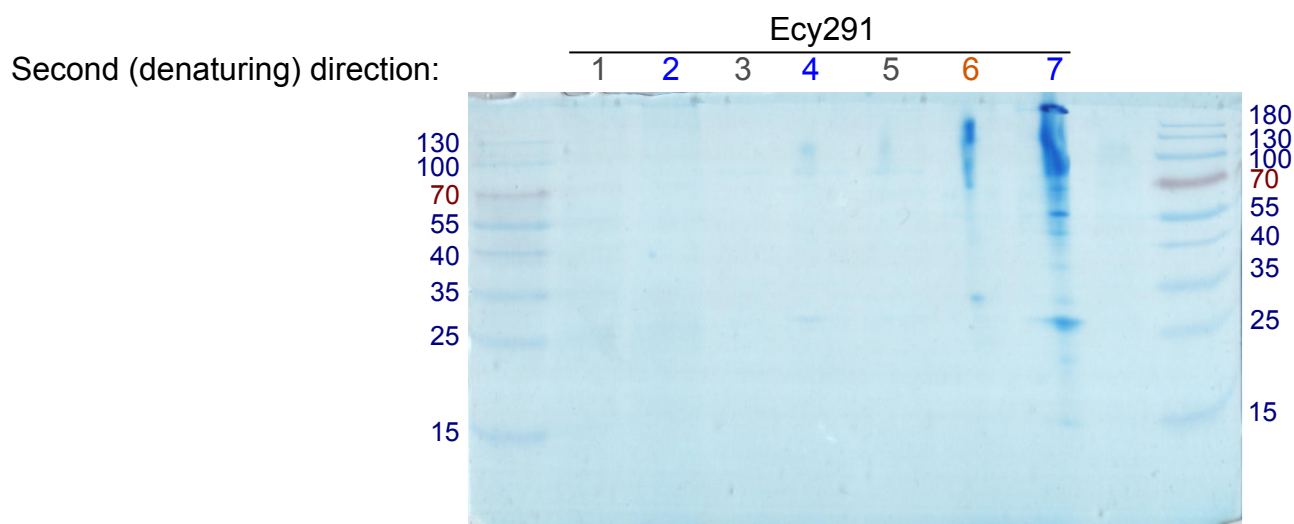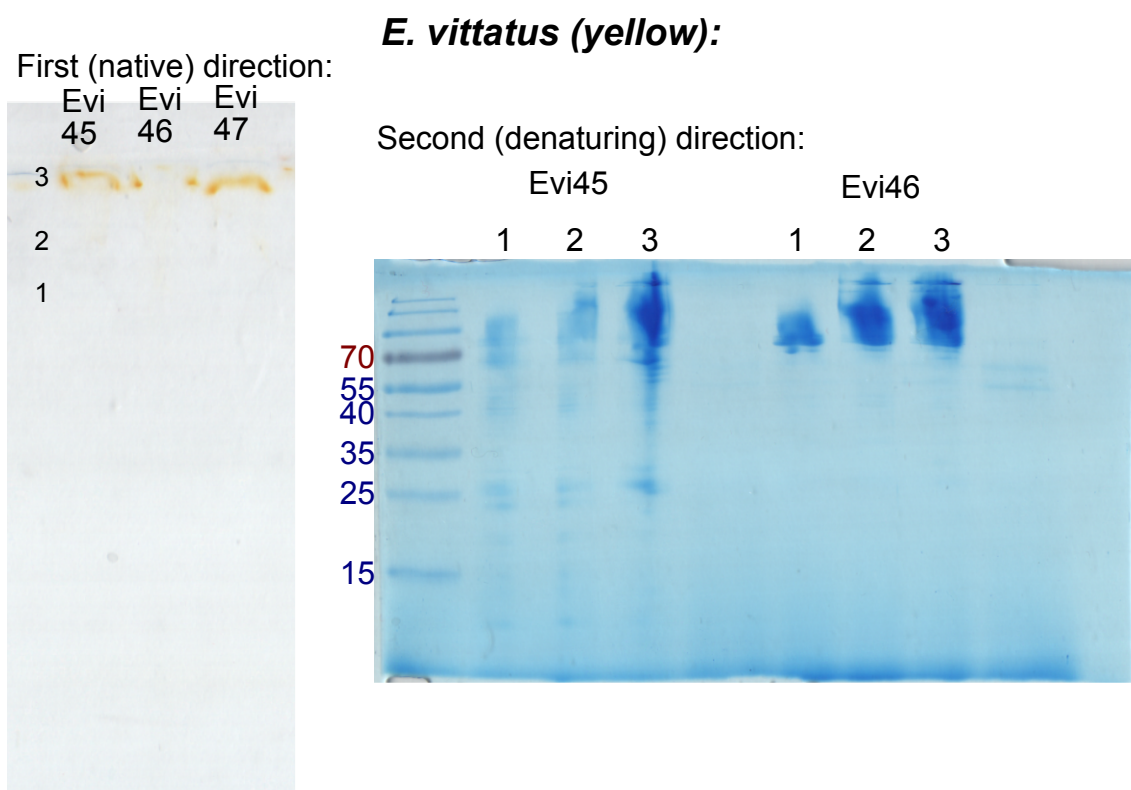

Supplement: Supplemental Information 5 — The PageRuler Prestained Protein Ladder, 10 to 180 kDa (Thermo Scientific, USA) was used in both native and denaturing gels, but its mobility in native gels is unknown. [file peerj-08-9387-s005.pdf]
